# A week in the life of the JACMP

**DOI:** 10.1002/acm2.12300

**Published:** 2018-02-28

**Authors:** Michael D. Mills

Specifically, the week is 27 January–2 February 2018, 3 days before this Editorial is due and 2 days before the Super Bowl. The January issue was published 2 weeks ago and the web activity has returned to normal. The AAPM completed 1 year with Wiley last 31 December 2017 and with one more month completed, it seems right to take a measure of our activity. That week we had visits from 14 129 unique IP addresses. Consider there are about 25 000 active medical physicists with clinical duties in the world; probably up to one quarter of these physicists visit the JACMP each week. Most of the others are likely either professionals or members of the public looking for technical information about radiology and radiation oncology physics practice. Also, we note that during that period, 6224 JACMP articles were downloaded.

How do our users get to the JACMP web site? These are the top sites that refer our users:

1. google/organic
2. (direct)/(none)
3. ncbi.nim.nih.gov/referral
4. scholar.google.com/referral
5. medphys.org/referral
6. baidu/organic
7. AAPM.org/referral
8. scholar.google.co.uk/referral
9. scholar.google.co.jp/referral
10. scholar.google.co.in/referral

This is not surprising, but the fact that the general search engines drive many visitors to the website is encouraging. One of the functions of the JACMP is worldwide outreach for the AAPM. By offering clinical articles without a paywall, all clinical medical physicists and all patients, who are imaged or receive radiation therapy, directly and immediately benefit from JACMP scholarly investigations. In turn, our worldwide users are invited to investigate the many benefits that can only be had with AAPM membership.

During this week, these were the top 10 JACMP articles downloaded:

1. AAPM Medical Physics Practice Guideline 8.a.: Linear accelerator performance tests
2. Minimizing dose variation from the interplay effect in stereotactic radiation therapy using volumetric modulated arc therapy for lung cancer
3. Automatic patient positioning and gating window settings in respiratory‐gated stereotactic body radiation therapy for pancreatic cancer using fluoroscopic imaging
4. AAPM‐RSS Medical Physics Practice Guideline 9.a. for SRS‐SBRT
5. Volumetric modulated arc therapy for total body irradiation: A feasibility study using Pinnacle3 treatment planning system and Elekta Agility^™^ linac
6. COMP report: CQPR technical quality control guidelines for treatment planning systems
7. Survey of 5 mm small‐field output factor measurements in Australia
8. Artificial intelligence will reduce the need for clinical medical physicists
9. AAPM Medical Physics Practice Guideline 5.a.: Commissioning and QA of Treatment Planning Dose Calculations—Megavoltage Photon and Electron Beams
10. Dose tolerance limits and dose volume histogram evaluation for stereotactic body radiotherapy

The AAPM Practice Guidelines have always been popular downloads. There are many Canadian Organization of Medical Physicists (COMP) reports the JACMP will be publishing over the next several months; we expected these documents to be quite popular as well.

Finally, we ask the question, where in the world are JACMP users located? The answer is the following, followed by the percentage of total users:

1. United States 30.2
2. China 6.6
3. United Kingdom 5.7
4. Japan 5.1
5. Canada 5.0
6. India 4.4
7. Germany 4.3
8. Australia 2.9
9. South Korea 2.6
10. France 2.2

The United States now represents slightly less than one‐third of JACMP web activity. JACMP submissions and published articles follow about the same geographic percentages as its web activity. About half of JACMP users are English speaking, and half come from non‐English speaking countries.

Consider what the AAPM has to offer the medical physicists of the world. There is a massive amount of published scholarship and technical reports within the AAPM archives dealing not only with clinical medical physics but also with the science, education, administration and management/professional science of medical physics. There is no other comparable archive anywhere that matches this history and breadth of information. Clinical physicists need not reinvent the wheel as they seek to establish clinical physics practice in the nations of the world. So, I suggest to all JACMP users everywhere that AAPM membership is a next logical step as you realize your calling and your life's work.

Finally, this past week the JACMP was challenged with a breach of publishing ethics. Wiley, the Associate Editor and I answered this challenge, but it raises this point: Membership in the AAPM requires the highest level of commitment to ethical behavior. AAPM documents on ethical behavior and practice are available on line. Before you apply for AAPM Membership, you should review these documents and commit yourself to their ethical requirements. You should do this because only by subscribing to ethics in professional behavior will we together realize the promise of a bright future for our profession.

You can find relevant AAPM policies on ethics here:

https://www.aapm.org/medical_physicist/ethics.asp

https://www.aapm.org/org/policies/details.asp?id=260&type=PP&current=true

You may find the AAPM categories of membership, benefits, and the process for application here:

https://www.aapm.org/memb/?tab=3#MemberAppPanel

https://www.aapm.org/memb/?tab=1

https://www.aapm.org/memb/prospect/apply.asp

So, let this be the day you align yourself with your calling and its ethical requirements. If it seems compelling to you, we invite you to become a member of the AAPM.

